# Reliability of low-field upright MRI-based assessment of the polypropylene mesh after sacrocolporectopexy

**DOI:** 10.1007/s10151-026-03340-6

**Published:** 2026-05-11

**Authors:** Mart C. P. Kortman, Julia Evers, Frank F. J. Simonis, Anique T. M. Grob

**Affiliations:** 1 Multi-Modality Medical Imaging Group (M3i), Techmed Centre, University of Twente, Enschede, The Netherlands; 2 Departement of Gynecology, Ziekenhuisgroep Twente, Hengelo, The Netherlands; 3 Faculty of Medicine, Rijksuniversiteit Groningen, Groningen, The Netherlands

**Keywords:** Annotation method, Magnetic resonance imaging, Polypropylene mesh, Reliability assessment, Sacrocolporectopexy, Upright imaging

## Abstract

**Background:**

Multicompartment pelvic organ prolapse (POP) has a negative impact on the patient’s quality of life. Abdominal mesh surgery, e.g., sacrocolporectopexy (SCRP), is favored to treat complex multicompartment POP. SCRP is known for its anatomical improvement, while functional improvement is only 70% for defecatory problems. To improve our understanding of the mesh’s function, a reliable assessment in a clinically relevant position is important. The aim of this study therefore is to test the reliability of a standardized analysis method on mesh trajectory and position.

**Method:**

A total of 25 patients treated with SCRP were included. Images were acquired with a 0.25T tiltable magnetic resonance (MR) system (G-Scan Brio, Esaote, Genoa, Italy). The mesh was annotated using three-dimensional (3D) slicer software. Annotations were performed by two observers, with different experience levels, blinded to the results of the other observer. Reliability was tested by means of the intraclass correlation coefficient (ICC) and the mean Euclidean distance (MED), with clinical relevance set at a MED difference of 10 mm.

**Results:**

There was excellent agreement between the two observers for all mesh points (ICC = 0.97) and the lowest agreement for the anterior mesh points (ICC = 0.86) and the lowest points of the anterior mesh (ICC = 0.76). No clinically relevant difference in MED was found for all mesh points (9 mm), the mesh points above the connection point (8 mm), and the posterior mesh points (9 mm).

**Conclusions:**

Upright mesh assessment is feasible and reliable on 0.25T magnetic resonance (MR) images, allowing for future mesh analysis.

**Trial registration:**

NL79717.091.21, 03–01-2022.

## Introduction

Pelvic floor disorders (PFD) can be categorized as anterior, apical, or posterior compartment disorders, depending on which organs are affected. Examples of PFD are pelvic organ prolapse (POP), rectal prolapse (RP), and urinary incontinence (UI). Multicompartment PFD is common, with reported prevalences of 34–48%, stressing the need for a multidisciplinary PFD treatment approach [1–3]. When conservative treatments such as pelvic muscle training and pessaries fail, surgical treatment is regularly chosen. At first, native tissue repair options are used, such as colporrhaphy, Manchester procedure, or sacrospinous hysteropexy, while mesh repair options, such as sacrocolpopexy (SCP), ventral mesh rectopexy (VMR), or sacrocolporectopexy (SCRP), are reserved for more complex or recurrent prolapse.

While the effect of native tissue POP repair has been studied extensively; focusing on clinical outcomes, physical examination, and imaging modalities [4–7], studies on the true effect of transabdominal pelvic mesh surgery for POP and RP are limited. Reported functional improvement after mesh surgery such as fecal incontinence (FI) and obstructed defecation syndrome (ODS) indicate a limited functional improvement rate of 60–70%, while the surgery has a high impact on the quality of life of a patient [8]. The limited amount of currently available imaging studies have solely studied the effect of standalone VMR or SCP [9, 10] not on combined SCRP. In these studies, clinically uncommon polyvinylidene fluor meshes coated with iron-oxide were used to ensure visibility on magnetic resonance (MR) images. Besides that, these studies focused on the postoperative behavior of the mesh and the best MR-sequences for imaging using regular supine 1.5T or 3T MR. Therefore, the understanding of the true interaction between prolapse and the inserted mesh is limited, as prolapse is known to be underestimated in this supine position [11, 12]. We hypothesize that this will inherently limit the assessment of any correlation to clinical outcome, since accurate understanding of the placement of the mesh, combined with the anatomical and patient-reported outcome, is essential in the understanding and ultimately improving mesh surgery outcome.

Before any studies on the interaction of the mesh (VMR, SCRP, or SCP) and POP can be executed, the reliability of the assessment of the clinically standard uncoated mesh trajectory needs to be determined. The aim of this study therefore is to define a standardized analysis method on mesh trajectory and position on the basis of 0.25T MR images after VMR, SCRP, and SCP and to test its reliability.

## Method

In this study, the upright 0.25T MR imaging data from a single center (Ziekenhuisgroep Twente (ZGT) hospital, Hengelo, the Netherlands) prospective longitudinal study in patients undergoing SCRP or VMR between December 2022 and June 2024 for (internal) RP was used. Patients were excluded when they were not eligible to undergo an MRI scan, unable to stand without assistance for 20 min, their hip circumference exceeded the receiver-coil circumference (size 52) or had insufficient knowledge of the Dutch language. All patients were ≥ 18 years and provided written informed consent. The study was approved by the medical ethics committee and registered as NL79717.091.21.

The SCRP procedures were performed by one gynecologist and one colorectal surgeon and the VMR procedures were performed by a colorectal surgeon. During both procedures, the DaVinci Xi system (Intuitive Surgical, Sunnyvale, CA, USA) was used for robotic assistance. The surgical technique has previously been described in detail by Kortman et al. [13] During surgery three 9-mm surgical clips were attached to the inferior end of the anterior and posterior mesh as markers.

The three-dimensional (3D) MRI volumes were acquired with a 0.25T tiltable MR-scanner (G-Scan Brio, Esaote, Genoa, Italy) located at the University of Twente, the Netherlands. Before scanning, contrast agents were inserted into the vagina and rectum of the patients: ultrasound gel and instant mashed potatoes, respectively. The patients were positioned in the MR scanner and scanned in supine and upright position. A multichannel spine coil was used for acquisition. The 3D hybrid contrast enhancement (HYCE) image data was acquired in the sagittal direction and used for annotation of the mesh. After 12 patients completed the first follow-up visit, the 3D HYCE acquisition parameters were slightly altered and the 3D dual-echo steady state free precession (SSFP) sequence was added to improve mesh visibility. The parameters of both sequences can be found in Table 1.

**Table 1:** Sequence parameters of the MR sequences used for analysis. During the course of the study, the 3D HYCE was improved, and the 3D dual-echo SSFP was added

Parameter	3D dual-echo SSFP sequence	3D HYCE sequence	
Sequence type	3D dual-echo steady state free precession	3D balanced steady state free precession	
Echo time	10 ms	4 ms	
Repetition time	20 ms	8 ms	
Flip angle	35°	60° or 35°	
Field of view	250 × 250 × 180 mm^3^	250 × 250 × 250 mm^3^	
Acquisition matrix	176 × 160 × 72	224 × 124 × 100 or 224 × 170 × 76	
Reconstructed resolution	1 × 1 × 1 mm^3^	0.5 × 0.5 × 0.5 mm^3^	
Number of averages	1	3	
Acquisition time	3:52 min	3:32 or 3:41 min	

**Fig. 1:**
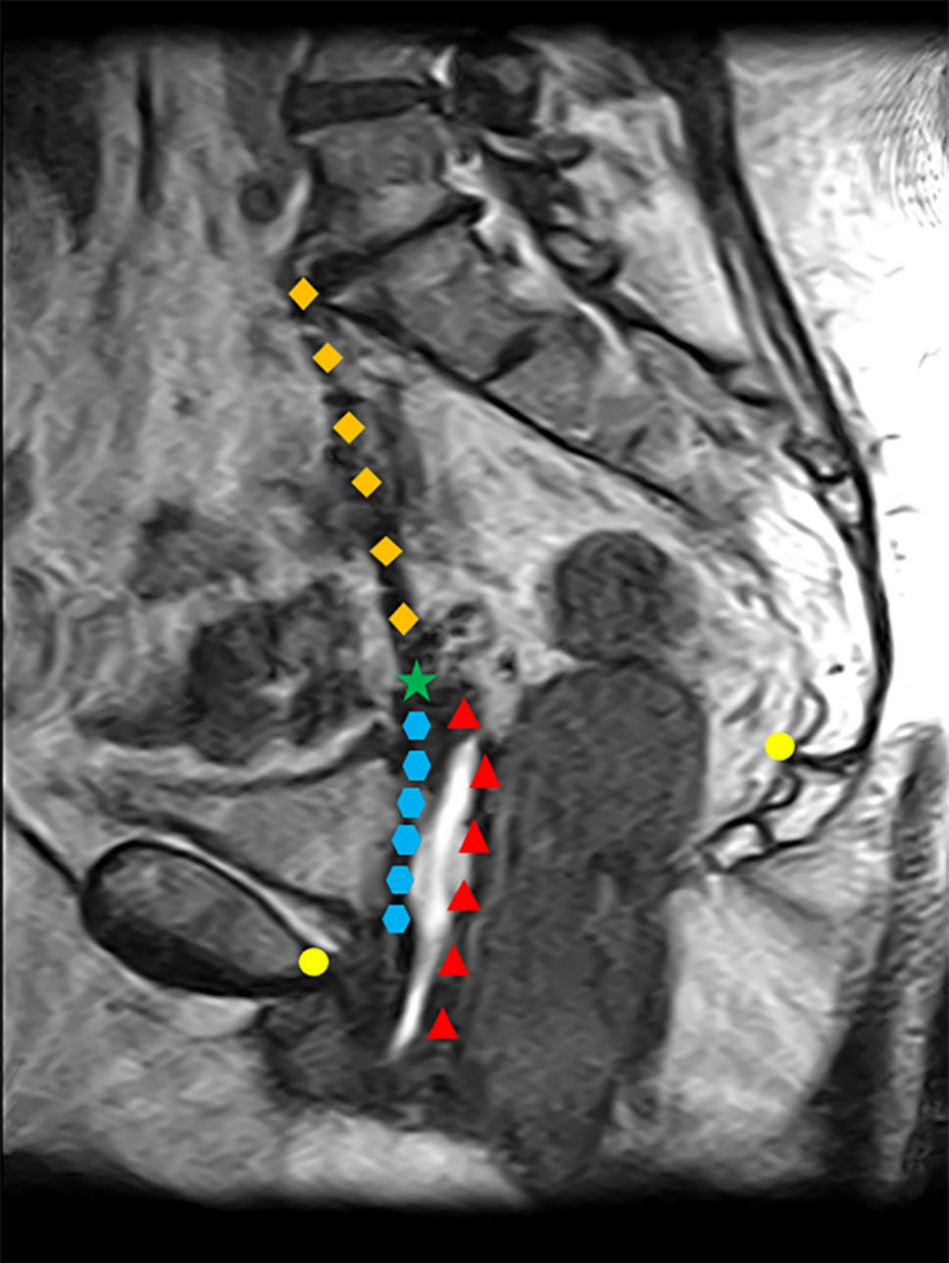
A midsagittal slice of a patient with vaginal and rectal contrast agents in upright position, in which the mesh and PICS plane are annotated. The yellow dots represent the PICS plane, the orange diamonds the top part of the mesh, the green star the mesh connection point, the blue hexagons the anterior mesh, and the red triangles the posterior mesh

For annotation of the mesh, the 3D slicer (version 5.8.1) was used [14]. At first, the pelvic inclination correction system (PICS) plane was defined by four anatomical points: the inferior pubic point (IPP), the sacrococcygeal joint (SCJ), and both sides of the ischial spine [15]. The IPP and SCJ are depicted in yellow on Fig. 1.

Then, the mesh connection (MC) point was annotated. The MC point is defined as the point where the anterior mesh is attached to the posterior mesh. In cases where the MC point was not identifiable, a point just above the vaginal vault was used. The mesh was divided into three parts: first, the part from the MC point to the anterior longitudinal ligament, second, from the MC point to the anterior lowest point, and third, from the MC point to the posterior lowest point. For each of the parts, seven points were placed equidistantly with the MC point as common point. In Fig. 1 the placement of the points is visualized: the green dot represents the connection point of the mesh, the orange points the top part of the mesh, the blue points the anterior part of the mesh, and the red points the posterior part of the mesh.

Two observers, with 3 years (MCPK) and less than 1 year (JE) experience in evaluating 0.25T MR images of the female pelvis applied the above described mesh annotation method on the upright MRI scans of 25 patients. The scans were evaluated within a timespan of 1 month in chronological order.

On the basis of an anticipated variation in visibility (and thus reliability) of the mesh on the different parts of the mesh (top part, anterior part, posterior part), the annotated points were pooled in different groups: all points, PICS points, mesh points above the MC point, anterior mesh points below the MC point, posterior mesh points below the MC point, lowest point of the anterior mesh, and lowest points of the posterior mesh. The interobserver reliability was determined by means of the intraclass correlation coefficient (ICC). The ICC was calculated with SPSS (version 28.0.1.0; IBM, Armonk, NY, USA) on the basis of a two-way mixed effect and absolute agreement. An ICC lower than 0.5 shows poor reliability, between 0.5 and 0.75 shows moderate reliability, between 0.75 and 0.9 shows a good reliability, and above 0.9 shows an excellent reliability [16]. While the ICC provides a clear picture on the agreement between the observers, it does not quantify it. To quantify the absolute error between the observers, the mean Euclidean distance (MED), in 3D, was computed. On the basis of the minimal difference that is required to affect the prolapse stage and have clinical implications, an absolute error of less than 10 mm was considered clinically irrelevant. For the purpose of comparison to previous studies the median Euclidian distance was calculated for the lowest point of the meshes and the connection point of the anterior and posterior mesh (green star in Fig. 1).

## Results

The upright 3D HYCE volumes of 25 patients were included, and the demographics are listed in Table 2.

**Table 2:** Demographics of the included patients (*n* = 25)

Age (mean (SD))	65 (± 9) years					
BMI (mean (SD))	26.3 (± 3.3)					
Preoperative POP-Q stage	Stage	0	1	2	3	4
	Anterior	3	6	13	1	1
	Middle	4	15	3	3	0
	Posterior	0	9	13	3	0
Parity	1:1 2:15 3:8 4:1					

Intraclass correlation coefficients for the different groups of points are listed in Table 3.

**Table 3:** Accuracy of mesh analysis by means of interobserver agreement represented as ICC values and mean Euclidean distance

Points	Intraclass correlation coefficient	Mean Euclidean distance (SD)
All points	0.97^*^	9 (± 8) mm
PICS points	0.98^*^	7 (± 13) mm
Mesh points above MC point	0.98^*^	8 (± 5) mm
Mesh points anterior below MC point	0.86^*^	12 (± 10) mm
Mesh points posterior below MC point	0.96^*^	9 (± 5) mm
Anterior lowest points	0.76^*^	17 (± 14) mm
Posterior lowest points	0.96^*^	11 (± 7) mm

^*^Statistically significant (*p* < 0.001)

There was excellent agreement between the two observers for: all points (0.97), the PICS points (0.98), the mesh points above the MC point (0.98), the posterior mesh points below the MC point (0.96), and the posterior lowest points (0.96). Lower ICC values defined as good agreement were found for the anterior mesh points below the MC point (0.86) and the lowest points of the anterior mesh points (0.76).

**Fig. 2:**
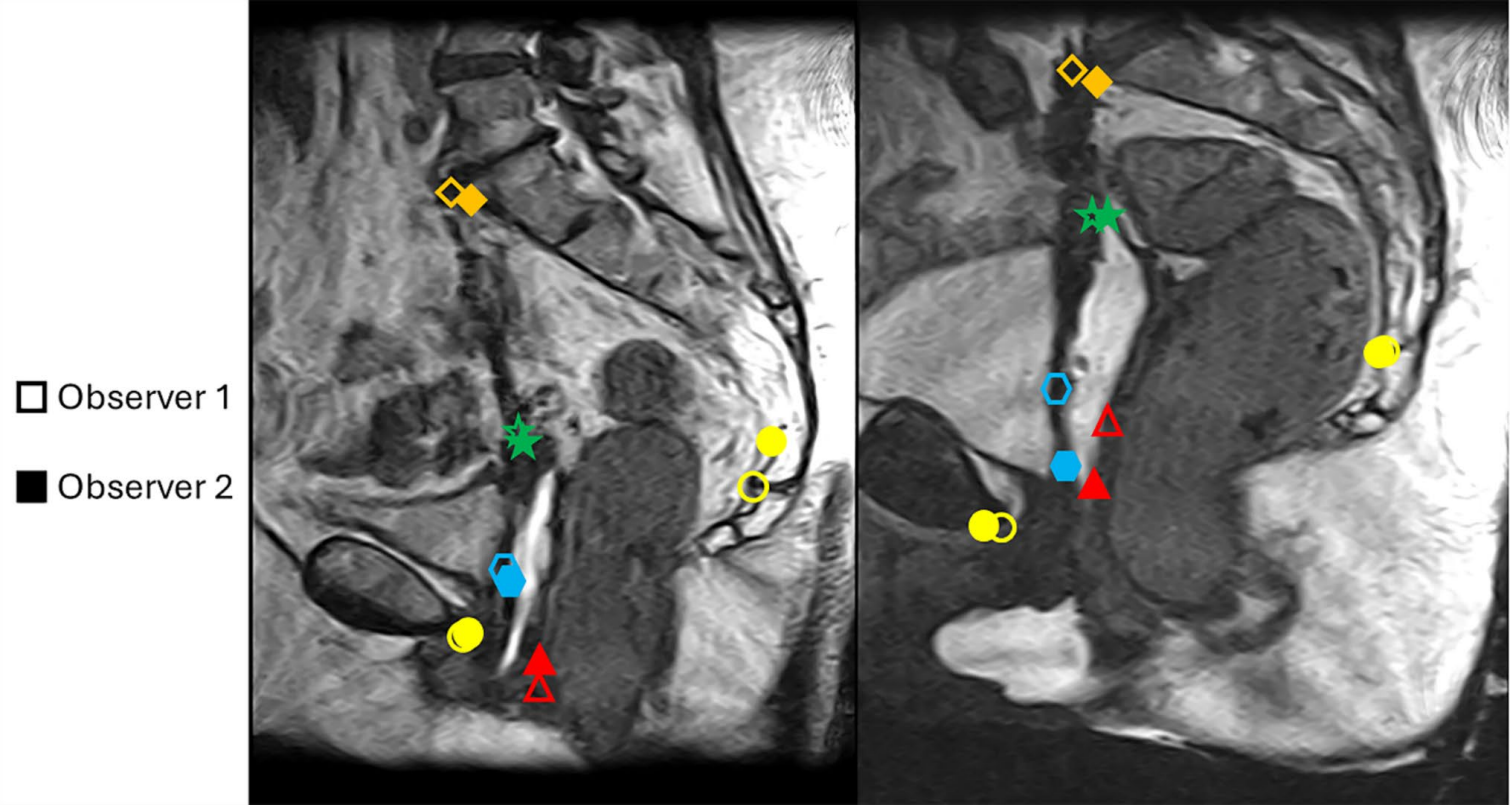
Two examples of midsagittal slices depicted with six key points annotated by both observers, the open figurines being placed by observer 1 and the filled figurines being placed by observer 2s. Yellow dots represent the inferior pubic point and sacrococcygeal joint, the green star illustrates the mesh connection point, the blue hexagon and red triangle represent the lowest points of the anterior and posterior mesh respectively, and the orange diamond indicates the mesh attachment to the anterior longitudinal ligament. Left: observer variation can be noticed on the sacrococcygeal joint. Right: observer variation is illustrated on the lowest point of the anterior and posterior mesh. For reference indication, the difference between these points is corresponding to the calculated MED

On the basis of the cut-off value of 10 mm for clinical relevance, there was no clinically relevant difference in point placement for the groups of all points (9 mm), PICS points (7 mm), mesh points above the MC point (8 mm), and posterior mesh points below the MC point (9 mm). A clinically relevant MED was found for the anterior mesh points below the MC point (12 mm) and both lowest points of the mesh (anterior: 17 mm and posterior: 11 mm). For the anterior lowest point, posterior lowest point, and MC point, the median Euclidean distance was calculated, being 12 mm, 11 mm, and 7 mm, respectively. In Fig. 2, an example of the difference between the observers is visualized.

## Discussion

The aim of this research was to define and evaluate the reliability of a standardized mesh annotation method after transabdominal pelvic mesh surgery on the basis of 0.25T MR images. Comparison of the annotations of two observers with different experience levels shows that the overall agreement is excellent, and there is no clinically relevant distance between the points placed by the observers. To the best of our knowledge, this is the first study describing a method and evaluating its reliability for the annotation of conventional (noncoated) polypropylene meshes after SCRP in a low field tiltable MR system. However, a detailed look into the different parts of the mesh indicated that the annotation of the lowest point of the anterior part of the mesh is less reliable and has a clinically relevant MED of 17 mm between the observers. This also applies, to a lesser extent, to the other annotated points of the anterior mesh. We hypothesize that the limited visibility of the mesh in the vesicovaginal and rectovaginal septum causes the large MED between the observers. In contrast to the mesh part between the MC point and sacral promontory, the mesh is hardly visible below mesh connection point, which can be attributed to the fact that the mesh is in a small tissue between organs filled with a substance that has a high signal density. This limitation was considered beforehand, resulting in the placement of 9-mm long surgical clips on the inferior end of the mesh. The clips result in metal artefacts on MR, which are in turn used to annotate the lowest point of the mesh. Owing to patient movement and the metal artifacts being narrow, the clips remain hard to locate, especially for novice observers. However, the calculated ICC for these most difficult mesh points is still above 0.75, which indicates good reliability [16]. We would like to argue that, owing to the inclusion of a novice observer, the currently proven reliability can be considered the lower limit. Therefore, the effect on the shape analysis is expected to be limited as the mesh follows the vesicovaginal and rectovaginal septa, which are clearly visible.

Comparing our low field upright mesh assessment to previous work was possible on the basis of the study by Sindhwani et al. [9] They assessed the mesh in 3D in 24 patients after laparoscopic SCP using 3T supine MRI and a mesh coated with iron oxide. The reported interobserver agreement was 0.97, which is similar to the overall reliability in this study. Therefore, a comparable, clinically irrelevant, median Euclidian distance for the connection point of the mesh was reported as 4 mm by Sindhwani et al. and 7 mm in this study. A difference was found in the median Euclidean distance for the lowest points of the anterior and posterior mesh, which was clinically irrelevant (3 and 6 mm) in the study by Sindhwani as compared with the clinically relevant different results in our study (11 and 12 mm). We hypothesize that this difference is likely attributed to the lower quality of the images and visibility of the mesh in our study, owing to a decreased signal-to-noise ratio. Additionally, Sindhwani et al. used iron-oxide-coated mesh implants, improving the visibility in the more challenging area of the vesicovaginal and rectovaginal septum. Despite the better MED reported by Sindhwani et al., we argue that the reliability of mesh assessment in both studies was good to excellent, indicating that a coated mesh is not mandatory for annotating the mesh toward obtaining a better understanding in its position and behavior.

The use of a low-field MR system yields strengths and weaknesses with respect to conventional MRI scanners. First of all, it offers the unique possibility to scan in upright position. Previous research shows that, in the context of POP, supine imaging results in an underestimation of the severity [11, 12]. Due to the use of a permanent magnet, the homogeneity of the field and, thereby, the effective field of view is reduced, but it remained sufficient to capture all relevant organs in one image. The main disadvantage of this low field system is the inherently lowered SNR. This was, however, compensated by increasing the scan time, leading to sufficient image quality for analysis, as is illustrated by our high ICC values.

A major strength of this study is the assessment of the reliability of mesh annotations on upright MRI scans, allowing for polypropylene mesh related studies in a more physiological position [11–13]. Despite using an MR system that is not available in most clinics, the insight gathered with upright imaging can aid in understanding the effect of SCRP. These findings can subsequently be translated into clinical practice without further need of the scanner. Another strength of this study was that the reliability for all parts of the mesh was good to excellent, while one observer had little training in recognizing mesh implants on MRI. Since training improves the observers assessment of MR images, as previously described in the field of endometriosis [17, 18], current study results suggests that after training a better (excellent) assessment of the mesh position can be performed.

A limitation of this study is the inability of visual conformation of the mesh in the rectovaginal and vesicovaginal septum, limiting accurate annotation, meaning that the observers positioned the points on the basis of visual inspection of the trajectory of the surrounding organs such as the bladder, vaginal wall and/or rectum rather than the mesh itself. Since the MC point and the lowest points of the mesh are known, and the mesh is directly attached to these organs, this indirect placement of points should result in sufficient match to the mesh trajectory. Despite being the main challenge of mesh trajectory assessment, ICC values indicate an excellent agreement for the mesh annotation in the vesicovaginal and rectovaginal septum, 0.86 and 0.96, respectively, and no clinical relevant error for the mesh annotation in the rectovaginal septum, i.e., 9 mm. Other limitations of this study are the small sample size and it being a single-center study, which poses a risk for selection bias. This should be addressed in future studies to allow for studies on the relation between mesh shape and clinical outcome.

Our study shows that there is no need to use expensive and clinically not-used MR-visible polyvinylidene fluoride meshes to assess the effect of SCRP. This proven reliability of non-iron-oxide-coated mesh assessment opens up a new field of research toward an understanding on the working mechanism of the mesh and the limited improvement in functional outcome. Additionally, we prove that mesh assessment is feasible on MR images acquired in the upright position and with a lower (0.25T) magnetic field. In future studies on mesh shape and placement, this method will be used for quantification. We argue that quantifying the location of the pelvic organs, the position and shape of the mesh as well as the mobility of the organs after mesh insertion is essential in further understanding the effect of VMR and SCRP. A dedicated clinical study on a large cohort of patients with MR measurements as well as questionnaires before surgery and at short and long term follow-up is strongly recommended.

## Data Availability

The data that support the findings of this study are not openly available and are available from the corresponding author upon reasonable request.
